# Real-life nanoplastics induce endothelial dysfunction in primary human endothelial cells

**DOI:** 10.1007/s00204-026-04417-9

**Published:** 2026-05-05

**Authors:** Joan Martín-Pérez, Michelle Morataya-Reyes, Aliro Villacorta, Claudia Anguita-Solé, Juan Francisco Ferrer, Irene Barguilla, Mohamed Alaraby, Ricard Marcos, Alba Hernández, Alba García-Rodríguez

**Affiliations:** 1 Group of Mutagenesis, Department of Genetics and Microbiology, Faculty of Biosciences, Universitat Autònoma de Barcelona, Campus of Bellaterra, 08193 Cerdanyola del Vallès, Barcelona, Spain; 2 Facultad de Recursos Naturales Renovables, Universidad Arturo Prat, Iquique, Chile; 3 Plastics Technology Center, AIMPLAS, Valencia Parc Tecnològic, 46980 Paterna, Spain; 4 Zoology Department, Faculty of Science, Sohag University, Sohag, 82524 Egypt

**Keywords:** Polystyrene, Polylactic acid, Polytetrafluoroethylene, Polyethylene terephthalate, Primary human umbilical vein endothelial cells

## Abstract

Understanding how nanoplastics (NPLs) exposure affects vascular endothelium is essential for determining their potential cardiovascular risk. To this end, four different NPLs of similar nominal sizes (about 200 nm), but different environmental relevance, have been used. They are: (i) spherical and monodisperse pristine polystyrene (PS), (ii) biodegradable polylactic acid (PLA), (iii) moderately irregular and polydisperse polytetrafluoroethylene (PTFE), and (iv) highly irregular and polydisperse polyethylene terephthalate (PET) derived from post-consumer bottles. To determine their hazardous risk, primary human umbilical vein endothelial cells (HUVECs) were used as a physiologically relevant model of the vascular endothelium. Results show that all NPLs were internalized by HUVECs, although uptake efficiency and intracellular distribution varied among polymers. None of the NPLs induced cytotoxicity or DNA damage at 25 µg/mL for 24 h. However, PTFE- and PET-NPLs elicited functional alterations consistent with endothelial dysfunction. PET-NPLs triggered IL-6 secretion and intracellular cholesterol accumulation, while both PTFE- and PET-NPLs significantly impaired cell migration, reducing wound closure. These findings reveal a clear gradient of biological impact, with irregular NPLs inducing stronger endothelial stress responses. By linking morphological realism to vascular inflammation, cholesterol dysregulation, and impaired migration, this study underscores the relevance of environmentally realistic NPLs into human health risk assessment frameworks.

**Supplementary Information:**

The online version contains supplementary material available at 10.1007/s00204-026-04417-9.

## Introduction

Plastics are synthetic organic polymers primarily derived from petroleum or other hydrocarbon sources, with some biodegradable variants produced from renewable resources such as cellulose or cornstarch (Nayanathara Thathsarani Pilapitiya and Ratnayake 2024). Their durability, lightweight, cost-effectiveness, and excellent barrier properties have driven their widespread use across numerous sectors, including the food industry (Ncube et al. 2021). In this context, plastics are employed in applications ranging from beverage bottles and packaging films to non-stick cookware and biodegradable tableware. During use, these materials can degrade through various physicochemical processes, such as mechanical abrasion, thermal stress, or prolonged contact with food and beverages, resulting in the environment release of microplastics (< 1 mm) and nanoplastics (< 1 μm), collectively referred to as micro- and nanoplastics (MNPLs).

In the environment, MNPLs enter the food web (Hussain et al. 2023), as documented for several polymer types, including polystyrene (PS) from disposable food containers and packaging, polylactic acid (PLA) from biodegradable packaging and teabags (Banaei et al. 2023), polytetrafluoroethylene (PTFE) from non-stick cookware (Cole et al. 2024), and polyethylene terephthalate (PET) from water bottles (Hussain et al. 2023). Once in the food chain, MNPLs can enter the human body primarily through ingestion, cross the intestinal barrier, and distribute to various tissues via systemic circulation (Rajendran and Chandrasekaran 2023). This is particularly relevant for nanoplastics, whose nanometric size allows for more efficient uptake and translocation across biological barriers, facilitating their biodistribution (Jahedi and Jaafarzadeh Haghighi Fard 2025). The ability of different MNPLs (such as those composed of PS, PLA, PTFE, and PET) to traverse biological barriers, including the intestinal barrier, has been demonstrated in several experimental models, suggesting their potential to reach the bloodstream (Alaraby et al. 2024; Landrigan et al. 2025). Despite the technical challenges associated with detecting MNPLs in complex biological matrices such as blood, Leslie et al. (2022) successfully identified PS- and PET-based MNPLs in human blood samples, providing direct evidence of systemic exposure. Once in circulation, MNPLs can interact with endothelial cells (ECs), which form the inner lining of blood vessels. ECs are key regulators of vascular homeostasis, controlling permeability, inflammatory signaling, and angiogenesis, and their dysfunction is strongly associated with the development of cardiovascular diseases (Xue et al. 2023). Several studies have reported associations between the presence of MNPLs in humans and adverse cardiovascular outcomes, suggesting a potential link between MNPL exposure and cardiovascular disease risk (Zhang et al. 2025; Prattichizzo et al. 2024). In this context, the interaction between MNPLs and endothelial cells may represent a key event underlying the development of such adverse cardiovascular effects.

In in vitro studies, human umbilical vein endothelial cells (HUVECs) are widely used as a physiologically relevant model, as they are primary human ECs derived from the umbilical vein and closely reproduce the structural and functional characteristics of the vascular endothelium (Cao et al. 2017). Studies using HUVECs have shown that PS-NPLs can induce a wide range of adverse effects, often modulated by particle charge and size. Reported impacts include reduced cell viability, increased production of reactive oxygen species (ROS), mitochondrial dysfunction, impaired autophagy, and activation of inflammatory pathways (Fu et al. 2022; Lu et al. 2022; Ahamed and Javed Akhtar 2024; Martín-Pérez et al. 2024).

Unfortunately, most research on the vascular effects of NPLs has focused on commercially available PS-NPL, mainly due to the limited availability of MNPL test materials (Drzewinska and Belz 2024). These pristine PS-NPL, are spherical, monodisperse, and chemically uniform, which facilitate experimental reproducibility; nevertheless, they fail to capture the diversity of morphologies, colloidal behaviors and surface chemistries present in the environmental MNPLs (Ma et al. 2024). Consequently, establishing links between MNPL exposure, endothelial responses, and downstream cardiovascular effects remains difficult without studies employing test materials that more closely mimic those found in real-life scenarios. To address this gap, the present study compares the biological effects of four different NPLs test materials (PS-NPLs, PLA-NPLs, PTFE-NPLs, and PET-NPLs) of approximately similar size (about 200 nm) to minimize size-related variability. Thus, PS-NPLs and PLA-NPLs present regular and spherical shapes; PTFE-NPLs exhibit moderate irregularity; and PET-NPLs were produced in-house from post-consumer water bottles, generating irregular, polydisperse particles that better resemble environmentally weathered plastics (Villacorta et al. 2022). With such four NPL types, internalization kinetics and multiple biological effects (including cytotoxicity and apoptosis, genotoxicity, inflammation, intracellular cholesterol accumulation, and cell migration) was investigated and comparatively assessed under standardized exposure conditions in HUVECs. By combining both commercially available and environmentally realistic NPLs within a controlled experimental framework, and using a low concentration, this work provides new insights into how polymer chemistry, particle morphology, and colloidal behavior influence endothelial cell responses, thereby contributing to a better understanding of the potential cardiovascular risks associated with NPL exposure.

## Materials and methods

### Sources and labeling of the selected NPLs

NPLs of around 200 nm were obtained from different sources. Polystyrene nanoplastics (PS-NPLs; PP-015-10, nominal size 0.19 μm) were purchased as an aqueous suspension from Spherotech Inc. (Lake Forest, IL, USA). Polylactic acid nanoplastics (PLA-NPLs; PRO20-0103-80) were produced by AIMPLAS (Valencia, Spain) in the frame of the EU project PlasticHeal, following the protocol previously described (Alaraby et al. 2024). Polytetrafluoroethylene nanoplastics (PTFE-NPLs; 430935-5G, nominal size 1 μm) in powder form were purchased from Sigma-Aldrich (St. Louis, MO, USA) and dispersed in Milli-Q water. Polyethylene terephthalate nanoplastics (PET-NPLs) were prepared in-house from commercial water bottles (Villacorta et al. 2022). Briefly, PET fragments from commercial water bottles were mechanically sanded using a diamond rotary burr to avoid metal contamination. The resulting debris was sieved (< 0.20 mm) and chemically dispersed in trifluoroacetic acid (TFA) at 50 °C under continuous stirring. After centrifugation, the pellet was resuspended in 0.5% sodium dodecyl sulfate (SDS) and subjected to ultrasonication. Following 1 h of sedimentation to remove larger particles, the upper fraction was collected, extensively washed (Milli-Q water and ethanol) to eliminate SDS residues, and resuspended in Milli-Q water. Finally, the suspension was sonicated, aliquoted, snap-frozen in liquid nitrogen, and stored at -80 °C. All NPLs were dispersed using the NanoGenotox protocol (Jensen et al. 2009). For internalization assays, NPLs were fluorescently labeled with iDye Poly Pink textile dye (Villacorta et al. 2024). Labeling was performed by incubating 1 mL of each NPL suspension (5 mg/mL) with 0.01 g of dye at 70 °C for 2 h. The mixture was diluted with 9 mL of Milli-Q water, and excess dye was removed by repeated centrifugation (Amicon^®^ Ultra-15 Ultracel^®^-100 K, Merck KGaA, Darmstadt, Germany) at 3500 rcf for 15 min, performed at least ten times. The retained fraction (80–160 µL) was recovered, diluted to 1 mL with Milli-Q water, aliquoted, and stored at 4 °C protected from light until use.

### NPLs characterization

For dry-state morphology, NPL suspensions in Milli-Q water (200 µg/mL) were deposited onto carbon-coated copper grids, air-dried overnight, and imaged with a JEOL JEM-1400 transmission electron microscopy (TEM) operated at 120 kV (JEOL Ltd., Tokyo, Japan). Micrographs were acquired using an Orius SC200D camera (Gatan, Ametek Inc., Berwyn, PA, USA), and Martin diameters of 100 individual NPLs were measured in ImageJ v1.8.0_172 to determine size distributions. For colloidal characterization, hydrodynamic size (dynamic light scattering, DLS) and surface charge (ζ-potential, electrophoretic light scattering) were measured with a Zetasizer^®^ Ultra (Malvern Panalytical, Cambridge, UK). Suspensions (100 µg/mL) were prepared in both Milli-Q water and Endothelial Cell Growth Medium-2 (EGM-2; PromoCell, Heidelberg, Germany). All measurements were conducted in triplicate.

### Cell line and culture conditions

Primary HUVEC (single donor, C-12200; PromoCell, Heidelberg, Germany) were used as a model of the human vascular endothelium. Cells were maintained in EGM-2 (C-22011; PromoCell, Heidelberg, Germany) in flasks coated with rat tail collagen I (5 µg/cm²; Corning, NY, USA) at 37 °C in a humidified 5% CO₂ atmosphere, with medium replaced every other day. Collagen coating was prepared by diluting collagen I in 20 mM acetic acid, incubating flasks for 1 h, and rinsing twice with PBS. Experiments were performed with cells up to passage 5.

### NPLs exposure

Cells were exposed to NPL suspensions prepared in EGM-2 medium at 25 µg/mL for 24 h, unless stated otherwise. Untreated cells cultured under the same conditions served as negative controls. Typically, cells were seeded at 26,300 cells/cm² in collagen I–coated wells (5 µg/cm²), grown for 48 h, and then the medium was replaced with the treatment for 24 h.

### NPLs internalization

Internalization of the different NPLs by HUVECs was assessed using flow cytometry, confocal microscopy, and TEM, providing complementary quantitative, qualitative, and ultrastructural information. Untreated cells were included as negative controls.

### Flow cytometry

Cells were exposed for 24 h to fluorescently labeled NPLs at 12.5, 25, or 50 µg/mL. After exposure, cells were washed twice with PBS, detached, and analyzed on a CytoFLEX cytometer (Beckman Coulter, Pasadena, CA, USA) using 561 nm excitation and 585/42 BP emission. A total of 10,000 events were collected per condition and analyzed with CytExpert software. Two parameters were quantified: (i) the percentage of cells positive for internalization and (ii) the mean fluorescence intensity per cell, reflecting the amount of internalized NPLs at each concentration. Side scatter (SSC) was also recorded to evaluate changes in cell complexity. Experiments were performed in triplicate, with technical duplicates for each condition.

### Confocal microscopy

For intracellular localization, HUVECs were seeded on collagen I–coated µ-Dish 35 mm plates (Ibidi GmbH, Gräfelfing, Germany), cultured for 48 h, and then exposed for 24 h to fluorescently labeled NPLs at 25 µg/mL. After exposure, cells were washed twice with PBS and incubated with fresh medium containing Hoechst 33,342 (1:500; nuclei) and CellMask™ Deep Red (1:500; plasma membrane) (Thermo Fisher Scientific). Imaging was performed on an LSM 980 Airyscan 2 equipped with a Plan-Apochromat 63× objective (Carl Zeiss Microscopy GmbH, Jena, Germany). Excitation/emission settings were 348/455 nm for nuclei, 659/676 nm for plasma membrane, and 577/603 nm for iDye Poly Pink-labeled NPLs. Several random fields were imaged per condition. Image processing was performed using Fiji/ImageJ (Schindelin et al. 2012).

### Transmission electron microscopy (TEM)

For ultrastructural confirmation, HUVECs were seeded in collagen I-coated T25 flasks, cultured for 48 h, and then exposed for 24 h to 25 µg/mL of non-labeled NPLs. After treatment, cells were washed with PBS, detached, and fixed in 2.5% glutaraldehyde and 2% paraformaldehyde in 0.1 M cacodylate buffer (pH 7.4). Samples were post-fixed with osmium tetroxide, dehydrated in graded acetone, embedded in Eponate 12™ resin (Ted Pella Inc., Redding, CA, USA), and polymerized at 60 °C. Ultrathin sections were obtained with an ultramicrotome, mounted on copper grids, contrasted with uranyl acetate and Reynolds lead citrate, and examined with a JEOL 1400 TEM (JEOL Ltd., Tokyo, Japan) equipped with an ES1000W Erlangshen CCD camera (Gatan Inc., Pleasanton, CA, USA). Several random fields were imaged. TEM processing steps followed conventional protocols as described (Annangi et al. 2015).

### Cell viability and apoptosis

Cell viability and apoptosis were assessed after NPLs treatment using the Dead Cell Apoptosis Kit with Annexin V Alexa Fluor 488 and Propidium Iodide (Thermo Fisher Scientific, Waltham, MA, USA) according to the manufacturer’s instructions. To avoid loss of detached apoptotic or dead cells, the culture supernatant was collected and combined with the trypsinized fraction before staining. After labeling, cells were analyzed by flow cytometry (CytoFLEX, Beckman Coulter, Pasadena, CA, USA) using 488 nm excitation with fluorescence emission collected at 525/40 BP and 690/50 BP. Gating was applied to distinguish live, apoptotic, and necrotic populations, and results were normalized to 100%. Camptothecin (5 µM, 24 h) was included as a positive control. Experiments were performed in triplicate, with technical duplicates for each condition.

### DNA damage induction

DNA damage levels were assessed using the comet assay with and without formamidopyrimidine DNA glycosylase (FPG) (Collins et al. 2023). The assay without FPG quantified DNA single-strand breaks (basal damage), whereas the FPG-modified assay measured the total DNA damage, including both single-strand breaks and oxidized DNA bases. After corresponding treatments, cells were washed with PBS, trypsinized, centrifuged (300 rcf, 8 min, 4 °C), and resuspended in cold PBS at 1 × 10^6^ cells/mL. A 1:10 dilution in 0.75% low-melting agarose (37 °C) was prepared, and 7 µL aliquots were placed on Gelbond^®^ films (GBF, Lonza Bioscience, Basel, Switzerland). Cells were lysed for 2 h at 4 °C (2.5 M NaCl, 0.1 M EDTA, 0.01 M Tris, 0.2 M NaOH, 1% Triton X-100, 1% N-lauroylsarcosine, 10% DMSO; pH 10), rinsed twice in enzyme buffer (0.04 M HEPES, 0.1 M KCl, 0.5 mM EDTA, 0.2 mg/mL; pH 8), and incubated for 50 min in the same buffer. A second incubation with or without activated FPG was carried out at 37 °C for 30 min. After washing in electrophoresis buffer (0.3 M NaOH, 1 mM EDTA; pH 13.4), DNA was allowed to unwind for 25 min before electrophoresis (20 V, 300 mA, 20 min). GBFs were rinsed in PBS, fixed in ethanol, and stained with SYBR Gold (1:2500 in TE buffer: 10 mM Tris Base, 1 mM EDTA; pH 8). Comets were visualized with an Olympus BX50 microscope (20×), and the percentage of DNA in the tail was quantified using Komet 5.5 software (Kinetic Imaging Ltd, Liverpool, UK). Two independent biological experiments were conducted, each with two technical replicates, and 100 cells were scored per replicate. Methyl methanesulfonate (MMS, 200 µM, 40 min) was used as a positive control for single-strand breaks, and potassium bromate (KBrO₃, 5 mM, 40 min) as a positive control for total DNA damage in the FPG-modified assay.

### IL-6 secretion levels

IL-6 levels in culture supernatants were measured after 24 h exposure to NPLs using the commercial IL-6 ELISA kit (KHC0061, Invitrogen; Waltham, MA, USA) according to the manufacturer’s instructions. Absorbance was read at 450 nm, and concentrations were calculated from standard curves generated by a four-parameter logistic model. Three independent experiments were performed, each with technical duplicates per condition.

### Intracellular cholesterol levels

HUVECs were seeded in µ-Plate 24 Well black plates (ibidi, Gräfelfing, Germany), and cholesterol levels were evaluated by Filipin III staining after 24 h exposure to NPLs. Briefly, cells were fixed with a two-step protocol: pre-fixation in a 1:1 mix of EGM-2 and 4% paraformaldehyde (PFA) for 10 min at 37 °C, followed by 4% PFA for 10 min at room temperature. After PBS washes, residual aldehydes were quenched with 1.5 mg/mL glycine in PBS. Filipin III (SAE0087, Sigma-Aldrich, Steinheim, Germany) was prepared in PBS supplemented with 10% FBS and applied at a final concentration of 0.05 mg/mL for 2 h at room temperature in the dark. Images were acquired with a Zeiss LSM 980 Airyscan 2 confocal microscope (Carl Zeiss Microscopy GmbH, Jena, Germany) using a 20× objective (excitation 353 nm, emission 465 nm). For image analysis, 100 independent cells (manual Regions of Interest) were quantified per technical replicate, resulting in a total of 600 cells across three biological experiments with two technical replicates each. Fluorescence was analyzed as the mean intensity of each ROI.

### Cell migration potential

Cell migration was assessed with a wound-healing assay using Culture-Inserts 2 Well (Ibidi GmbH, Gräfelfing, Germany). Inserts were placed in collagen-coated wells, and 21,000 cells were seeded in each compartment in EGM-2 medium containing 25 µg/mL of NPLs. After 24 h (37 °C, 5% CO₂), inserts were removed, wells washed, and fresh medium with the same NPL concentration added. Images were acquired at 0 h and every 2 h up to 8 h with a Zeiss Axio Observer A1 inverted microscope (10× objective; Carl Zeiss Microscopy GmbH, Jena, Germany). Wound closure was analyzed in ImageJ (v1.8.0_172) with the “Wound Healing Size Tool” plug-in (Suarez-Arnedo et al. 2020). Two parameters were calculated: (i) front velocity, expressed in µm/h, as (A_0_−A_*t*_)/(2·*L*·Δ*t*), and (ii) percentage of wound closure, as (A_0_−A_*t*_)/A_0_ × 100. Here A_0_​ and A_*t*_​ are wound areas at 0 h and time *t*,* L* is wound length, and Δ the elapsed time. Four independent experiments were performed, each with technical triplicates.

### Statistical analysis

Data analysis was performed with GraphPad Prism 9 (GraphPad Software Inc., CA, USA). Normality was assessed using the Kolmogorov-Smirnov test. For normally distributed data, one-way ANOVA was followed by: Dunnett’s test (comparisons of each NPL vs. the negative control, excluding the positive control), Tukey’s test (pairwise comparisons among NPLs, excluding both the negative and positive control), and Dunnett’s test for the comparison of the positive control vs. the negative control (including all groups). For non-parametric data, the Kruskal-Wallis test with Dunn’s post hoc test was applied following the same criteria. Statistical significance was defined as *p ≤ 0.05, **p ≤ 0.01, and ***p ≤ 0.001. Results are expressed as mean ± SEM.

## Results and discussion

### NPLs characterization

**Fig. 1:**
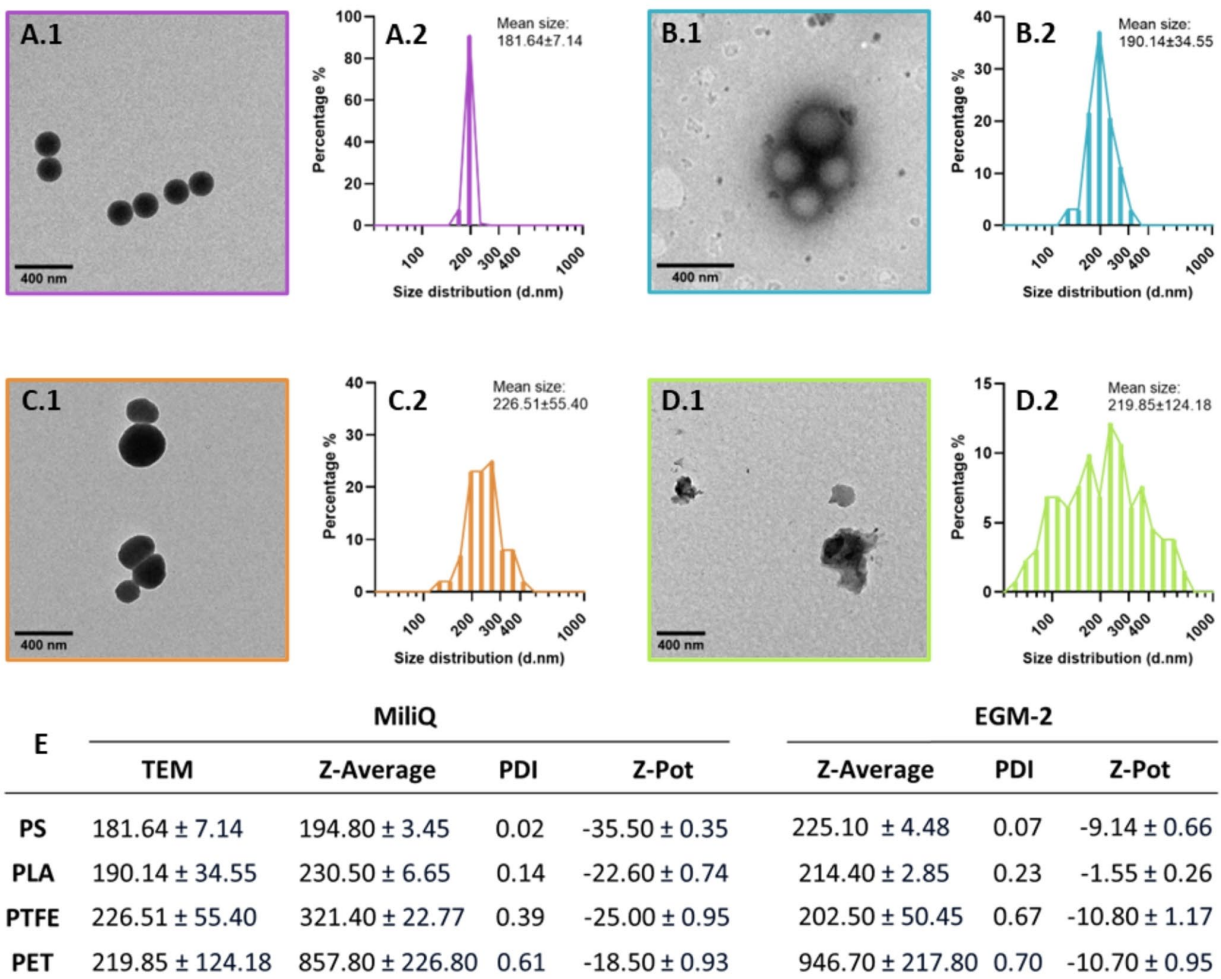
Characterization of NPLs. (A–D) TEM characterization in Milli-Q water for **A** PS, **B** PLA, **C** PTFE, and **D** PET. (**A.1**–**D.1**) Representative TEM images and (**A.2**–**D.2**) corresponding size distribution histograms obtained from TEM measurements. (**E**) Summary of TEM particle diameters, hydrodynamic diameters (DLS), and ζ-potential values of the NPLs in Milli-Q water and in EGM-2 medium. Sizes are expressed in nm (diameter) and ζ-potential in mV (mean ± SD)

TEM confirmed that all NPL types displayed mean diameters around 200 nm (Figs. 1A.2–D.2, E). This suggests that the biological effects observed in subsequent assays are likely influenced primarily by the intrinsic properties of each polymer and to differences in morphology and colloidal behavior, rather than by major size discrepancies between individual NPLs. Controlling for particle size is important, as it is a key factor affecting NPL-cell interactions and biological responses (Lu et al. 2022). In terms of morphology (Figs. 1A.1–D.1), PS-NPLs and PLA-NPLs presented uniform, spherical shapes, whereas PTFE-NPLs appeared spherical but slightly irregular. PET-NPLs displayed the most heterogeneous and irregular morphologies, consistent with Villacorta et al. (2022) observations. This gradation, from idealized synthetic spheres to irregular, “real-life” fragments, provides a model system spanning from controlled laboratory particles to those more representative of environmental NPLs (El Hadri et al. 2020). The same pattern was reflected in size distributions, PS-NPLs showed the narrowest range (164.9–200.7 nm; SD 7.1 nm), PLA-NPLs and PTFE-NPLs were broader (171.6–353.2 nm and 118.3–387.2 nm, respectively), and PET-NPLs displayed by far the widest dispersion (56.9–587.8 nm; SD 124.2 nm).

DLS measurements further characterized the NPLs in suspension (Fig. 1E). Hydrodynamic diameters in Milli-Q water were consistently larger than TEM sizes, as expected for hydrated particles (Wilson and Prud’homme 2021). Polydispersity indexes (PDI) values increased in EGM-2 compared to Milli-Q water across all polymers, consistent with biomolecule adsorption (protein corona formation) and subsequent particle aggregation in biological media (da Silva et al. 2019; Ferreira et al. 2022). PS-NPLs and PLA-NPLs maintained low PDI values (< 0.3), reflecting higher colloidal stability, whereas PTFE-NPLs and particularly PET-NPLs showed markedly higher values. The strong aggregation tendency of PET-NPLs was evident in its large hydrodynamic diameter (946.7 nm in EGM-2) and high PDI (0.70 in EGM-2), underscoring its resemblance to environmentally derived NPLs. ζ-potential values were negative in Milli-Q water (–18.5 to − 35.5 mV), reflecting stable dispersions, but shifted towards neutrality in EGM-2 (–1.6 to − 10.7 mV) for all NPLs. PLA-NPLs showed the strongest shift, reaching near-neutral values (–1.55 mV) in EGM-2, consistent with previous reports of PLA-NPLs charge neutralization in culture media (da Silva et al. 2019). The other polymers stabilized around − 10 mV in EGM-2, consistent with values reported for NPLs such as PS-NPLs dispersed in cell culture media (Martín-Pérez et al. 2024). This general reduction in surface charge is explained by protein adsorption on the NPL surface, which masks the original charge, reduces electrostatic repulsion, and facilitates aggregation in culture environments (da Silva et al. 2019); however, although these medium-dependent shifts are consistent with protein corona formation in biological media, we did not directly visualize or profile the corona after cellular internalization in this study.

Overall, TEM and DLS analyses indicate that while PS-NPLs and PLA-NPLs behave as relatively stable, uniform model systems, PTFE-NPLs shows limited stability, and PET-NPLs display strong aggregation and heterogeneity, making it the closest proxy for environmentally derived NPLs. Taken together, this panel provides a controlled gradation of environmental relevance, from idealized spherical reference particles (PS) to more heterogeneous and colloidally unstable materials (PTFE), real-life PLA spherical shaped resulting from the in-house degradation of PLA pellets, and ultimately to real-life, polydisperse PET fragments derived from PET water bottles.

### NPLs internalization

The uptake of the different NPLs was assessed using flow cytometry, confocal fluorescence microscopy, and TEM. Flow cytometry was performed at 12.5, 25, and 50 µg/mL to evaluate internalization across a concentration range. This approach identified polymer-dependent uptake patterns and guided the selection of optimal concentration for subsequent assays, ensuring substantial internalization while avoiding cellular overload and maintaining relatively lower concentrations compared to our previous studies. Choosing a concentration that preserves differential uptake among polymers is particularly valuable, as it enables comparison of their internalization patterns and how differences in uptake correlate with subsequent biological responses. Finally, at 25 µg/mL, confocal microscopy was applied to corroborate the intracellular distribution of NPLs, while TEM was used to provide ultrastructural insight into their localization.

### NPLs internalization by flow cytometry

**Fig. 2:**
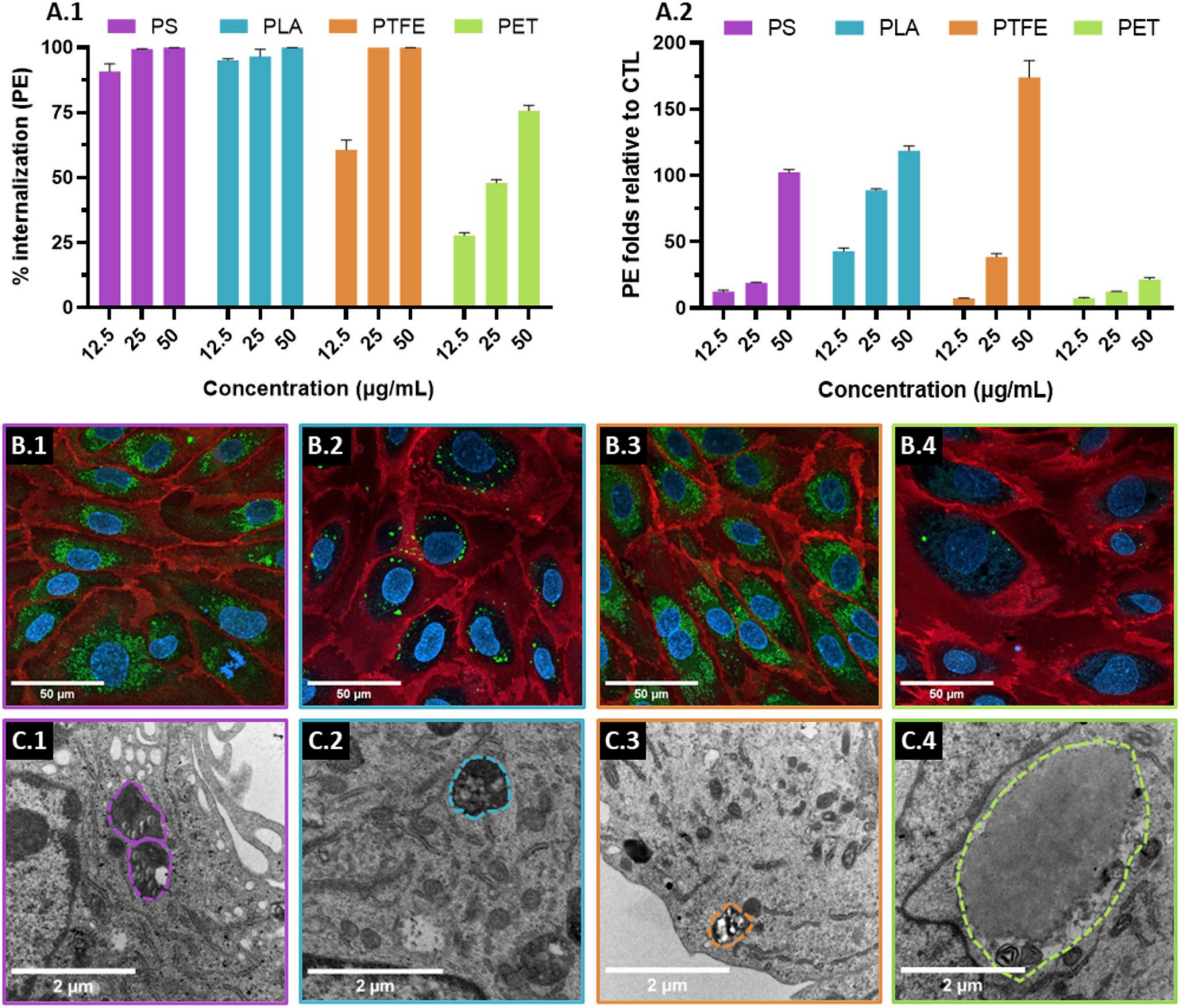
Internalization of NPLs. (**A.1**–**A.2**) Flow cytometry analysis of NPL uptake at 12.5, 25, and 50 µg/mL for 24 h: (**A.1**) percentage of NPL-positive cells and (**A.2**) mean fluorescence intensity of internalized NPLs. (**B.1**–**B.4**) Representative confocal images showing NPL uptake at 25 µg/mL for 24 h. NPLs are shown in green, nuclei in blue, and cell membrane in red. Panels correspond to (**B.1**) PS- NPLs, (**B.2**) PLA-NPLs, (**B.3**) PTFE-NPLs, and (**B.4**) PET-NPLs. (**C.1**–**C.4**) Representative TEM images of HUVECs exposed to 25 µg/mL of NPLs for 24 h. NPL-containing vesicles are highlighted with dotted lines. PS (**C.1**) formed numerous vesicles of 700–800 nm in diameter containing individual NPLs abundantly distributed throughout the cytoplasm. PLA (**C.2**) displayed less abundant vesicles of 800 nm containing clearly visible nanoparticles (zoom-in at Sup. Fig. 2 A.1–A.2), together with occasional larger vesicles up to 2 μm in diameter with granular content (Sup. Fig. 2 A.3). PTFE-NPLs (**C.3**) accumulated in numerous vesicles of 700–800 nm, each containing clearly individualized nanoparticles. PET-NPLs (**C.4**) induced scarce but very large vesicles, up to 4 μm in diameter, with diffuse and homogeneous electrodense internal content lacking discernible NPLs. Negative controls for confocal and TEM shown in Sup. Fig. 3

Flow cytometry after 24 h of exposure revealed generally high levels of NPL internalization across all polymers in HUVECs (Fig. 2A). This robust uptake underscores the suitability of HUVECs as a sensitive model for NPLs hazard assessment studies, in agreement with previous reports using nanomaterials (Cao et al. 2017). Compared to other cell types commonly used in nanotoxicology, such as Raji-B, THP-1, or TK6, which typically show < 10% of positive cells for 200 nm PS-NPLs even at 100 µg/mL (Tavakolpournegari et al. 2023), HUVECs demonstrated near-complete internalization at considerably lower exposure levels (12.5–50 µg/mL). This strong endocytic capacity has been documented in HUVECs for other nanomaterials, including silica nanoparticles, titanium dioxide, carbon nanotubes, and PS-NPLs (Blechinger et al. 2013; Gholinejad et al. 2019; Zhao et al. 2019; Martín-Pérez et al. 2024). To our knowledge, however, this is the first study to quantify the uptake of PLA-NPLs, PTFE-NPLs, and PET-NPLs in this endothelial cell model.

Despite the generally high internalization, uptake efficiency differed between polymers. PS-NPLs and PLA-NPLs showed nearly identical profiles, with approximately 100% of positive cells across all tested concentrations. PTFE-NPLs showed intermediate uptake at 12.5 µg/mL (60.7%) but reached complete internalization at 25 and 50 µg/mL, aligning with PS-NPLs and PLA-NPLs at these concentrations. In contrast, PET-NPLs consistently exhibited the lowest uptake, with only 27.8% of positive cells at 12.5 µg/mL, 48.1% at 25 µg/mL, and 75.8% at 50 µg/mL. Thus, while PS-NPLs, PLA-NPLs, and PTFE-NPLs converge toward near-complete internalization from 25 µg/mL onwards, PET-NPLs remain clearly less internalized. The reduced uptake of PET-NPLs is likely related to their colloidal instability. Dynamic light scattering of PET-NPLs suspensions in EGM-2 revealed the highest polydispersity index (0.70) and largest hydrodynamic size (946 ± 217.80 nm), indicating extensive aggregation. Since clathrin- and caveolin-mediated endocytosis generally accommodate nanoparticles up to 200 nm (Voigt et al. 2014; Sabourian et al. 2020), aggregates approaching or exceeding 500 nm are more dependent on macropinocytosis or phagocytosis, which are comparatively slower and less efficient internalization routes (Kuhn et al. 2014). In addition, polymer-specific surface chemistry and the resulting protein corona could modulate receptor interactions and influence uptake efficiency (Sousa De et al. 2021; Cao et al. 2022).

Mean fluorescence intensity (MFI; folds relative to control, Fig. 2B) supported these internalization patterns by reflecting intracellular accumulation. All polymer NPLs showed a clear dose-dependent increase, yet PET-NPLs consistently produced the lowest signal, with differences versus other polymers widening at higher concentrations. PTFE-NPLs exhibited lower MFI than PS-NPLs and PLA-NPLs at 12.5 µg/mL, but equaled or exceeded their levels at 25 and 50 µg/mL. The strong agreement between uptake percentages and MFI reinforces the conclusion that PET-NPLs are the least efficiently internalized polymer, while PS-NPLs, PLA-NPLs, and PTFE-NPLs are readily taken up. Based on these results, 25 µg/mL was selected as the working concentration for subsequent experiments. This concentration ensures robust uptake across polymers without causing cellular overload and remains within environmentally relevant ranges.

### NPLs internalization by confocal microscopy

Z-stack confocal imaging confirmed that the fluorescent signal corresponded to NPLs internalized within the cytoplasm rather than adhered to the plasma membrane, in line with previous reports using this approach (Morataya-Reyes et al. 2025a, b a). After 24 h of exposure at 25 µg/mL, most treatments produced a broad cytoplasmic distribution of NPL-associated fluorescence (Fig. 2B and supplementary Fig. S3), consistent with the quantitative flow cytometry data. In PS-NPLs-treated cells (Fig. 2B.1), fluorescence was homogeneously distributed throughout the cytoplasm of all cells, consistent with the complete proportion of NPL-positive HUVECs detected by flow cytometry. PLA-NPLs-treated cells (Fig. 2B.2) also showed 100% positivity, although with a less uniform and abundant cytoplasmic distribution. PTFE-NPLs-treated cells (Fig. 2B.3) resembled PS-NPLs, with fluorescence widely distributed across the cytoplasm of all cells. By contrast, PET-NPLs-treated cells (Fig. 2B.4) displayed a restricted uptake, with fluorescence confined to discrete punctate regions in only about half of the cell population, in line with the approximately 50% internalization measured by flow cytometry. Taken together, these fluorescence patterns demonstrate that both the magnitude of uptake and the subcellular distribution of NPLs depend strongly on the polymer type, emphasizing the importance of these differences when assessing NPL-cell interactions and their potential biological consequences.

### NPLs internalization by TEM

TEM corroborated the confocal observations and provided ultrastructural insights into the vesicular compartments containing NPLs (Fig. 2C, and supplementary Fig. S3).

In PS-NPLs-treated cells (Fig. 2C.1), numerous 700–800 nm vesicles containing individualized electron-dense nanoparticles were widely dispersed throughout the cytoplasm, consistent with lysosomal or autophagosomal compartments previously reported for PS-NPLs in RBL-2H3 cells and HUVECs (Liu et al. 2021; Lu et al. 2022 a). The presence of NPLs in these compartments agrees with receptor-mediated endocytosis via clathrin or caveolin (Liu et al. 2021). The high vesicular abundance matched the increase in complexity detected by flow cytometry side-scattering (SSC) (Supplementary Fig. S1). Compared to PS, PLA-NPLs exposure (Fig. 2C.2) produced fewer vesicles of similar size together with occasional larger vesicles (up to 2 μm) with granular content, suggesting partial involvement of macropinocytosis or phagocytosis-like uptake. Their less negative ζ-potential (− 1.55 mV vs. − 10 mV for other polymers) supports the formation of a distinct protein corona that may alter receptor interactions and promote alternative entry routes (Cao et al. 2017; Sousa De et al. 2021). PTFE-NPLs (Fig. 2C.3) showed a pattern like PS, with 700–800 nm vesicles containing clearly visible nanoparticles, yet their number was lower than in PS-treated cells. This morphology remains compatible with efficient clathrin- or caveolin-mediated endocytosis but indicates a slightly reduced extent of vesicular formation compared to PS. In contrast, PET-NPLs were in much larger, but scarce, vesicles (up to 4 μm in diameter), consistent with macropinosomes or phagosome-like structures formed through macropinocytosis or phagocytosis-like uptake in non-professional phagocytes such as HUVECs. This is in line with the hydrodynamic size measured by DLS for PET-NPLs in EGM-2, around 900 nm, which revealed extensive aggregation and supports uptake through these pathways. Since both macropinocytosis and phagocytic pathways eventually converge into the lysosomal route, the vesicles observed here may represent phagolysosomes. Such a mechanism agrees with the macropinocytosis-mediated internalization described by Liu et al. (2021) for 500 nm PS-NPLs and with the typical size ranges reported for phagocytosis in the literature (Sousa De et al. 2021). Although endocytic routes dominate, passive translocation across the plasma membrane cannot be ruled out for any of the tested materials, as hydrophobic and van der Waals interactions may allow limited diffusion of small NPLs (Liu et al. 2021). The low electron density of polymers, however, limits direct visualization of individualized NPLs in the cytoplasm by TEM (Sawyer et al. 2008). Overall, TEM confirms that polymer chemistry and colloidal behavior govern both the efficiency and pathway of NPL internalization in HUVECs.

### Cytotoxicity and genotoxicity

#### Cytotoxicity and apoptosis

**Fig. 3:**
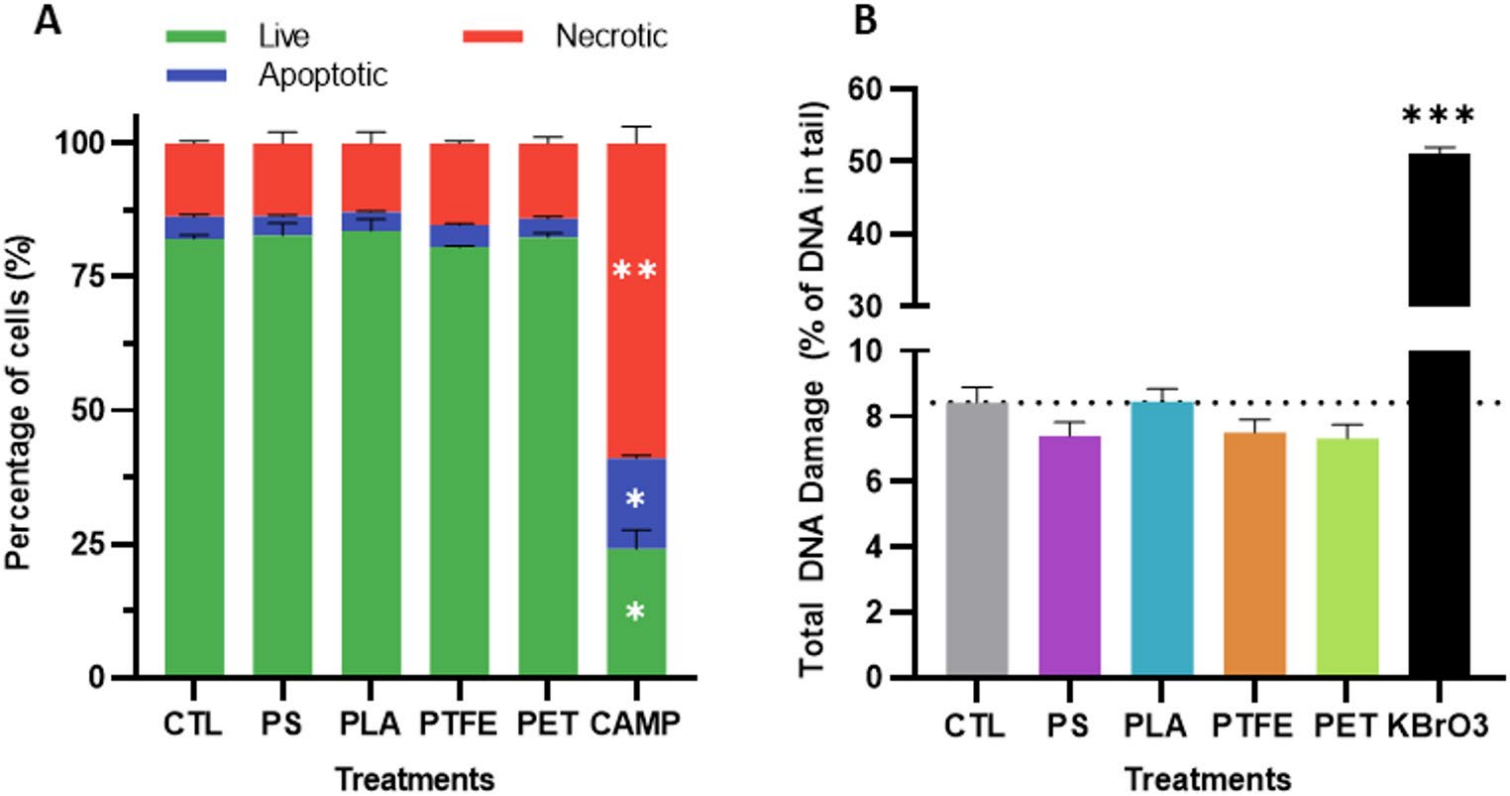
Cytotoxic and genotoxic effects of NPLs in HUVECs. **A** Apoptosis was assessed after 24 h exposure to NPLs (25 µg/mL) using Annexin V/PI staining and flow cytometry. Bars represent the percentage of viable (green), apoptotic (blue), and necrotic (red) cells. Camptothecin (5 µM, 24 h) was used as a positive control. **B** Total DNA damage was evaluated by the FPG-modified comet assay, expressed as the percentage of DNA in the tail. Data are expressed relative to the negative control (CTL, set to 1). KBrO₃ (5 mM, 40 min) served as positive control. Data are shown as mean ± SEM from ≥ 2 independent experiments. Statistical analysis was performed using the Kruskal–Wallis test with Dunn’s multiple comparisons. Significant differences were observed only between the positive controls and the negative control; no significant differences were detected for NPLs vs. control or among NPLs. *p ≤ 0.05, **p ≤ 0.01, ***p ≤ 0.001

The Annexin V/PI assay demonstrated that none of the NPL exposures compromised HUVEC viability at 25 µg/mL after 24 h (Fig. 3A). In all NPL-treated groups, more than 80% of cells remained viable, with necrotic fractions consistently below 15% and apoptotic fractions below 5%, values comparable to the negative control. These findings indicate that, under the tested conditions, NPLs of different polymer types do not trigger acute cytotoxicity or apoptosis in primary endothelial cells. In contrast, the apoptotic inducer camptothecin (5 µM, 24 h) produced the expected marked decrease in cell viability (< 25%) together with a pronounced increase in both necrotic (> 58%) and apoptotic (> 17%) populations, thereby validating the sensitivity of the assay (Morris and Geller 1996). These results are consistent with previous studies reporting a lack of acute cytotoxicity for PS-NPLs, PLA-NPLs, PTFE-NPLs, and PET-NPLs when tested at environmentally relevant concentrations across different cell types (Annangi et al. 2023; Banaei et al. 2023; Martín-Pérez et al. 2024; Abass et al. 2025).

The absence of cytotoxicity at the selected 25 µg/mL for 24 h validates this exposure condition as an appropriate experimental window to investigate more subtle environmentally meaningful endpoints, including genotoxicity, inflammatory signaling, metabolic disruption, and alterations in endothelial functionality, without confounding effects derived from cell death.

#### Genotoxicity

Although genotoxic effects of PS-NPLs have been reported in several human cell models, including HUVECs, Raji-B, and TK-6 cells (Rubio et al. 2020; Martín-Pérez et al. 2024), systematic evaluations encompassing other NPL types remain scarce. To date, no studies have examined the genotoxic potential of PLA-, PTFE-, or PET-NPLs in endothelial cells, and only limited evidence exists in other human cell systems. To address this gap, the comet assay was employed to assess whether the tested NPLs induce DNA strand breaks or oxidative DNA lesions. This assay has been widely validated as a reliable method for evaluating the genotoxicity of NPLs (García-Rodríguez et al. 2019).

In our study, application of the comet assay, both in its standard alkaline version and in the FPG-modified form to reveal oxidized bases, showed no increase in DNA damage levels in HUVECs exposed to 25 µg/mL of the different NPLs for 24 h (Fig. 3B and supplementary Fig. S4). Across all polymer treatments, values were about 6–8% DNA in tail, comparable to the negative control, with no significant differences observed. In contrast, the positive controls, potassium bromate (KBrO₃) for the FPG assay and methyl methanesulfonate (MMS) for the alkaline assay, produced the expected marked increases, confirming the assay sensitivity. Thus, under the tested conditions, 200 nm NPLs did not induce detectable single strand breaks or oxidative DNA lesions in primary endothelial cells. These results expand the scarce evidence on NPL-induced genotoxicity in endothelial cells and suggest that, under environmentally relevant conditions, these NPLs do not inherently compromise DNA integrity. Nonetheless, interpretation must remain cautious, as genotoxic responses to NPLs appear highly dependent on experimental context. For example, strand breaks have been detected with smaller PS NPLs in HUVECs (Martín-Pérez et al. 2024). PLA-NPLs-induced genotoxicity has been observed only at higher doses and longer exposures (García-Rodríguez et al. 2024; Morataya-Reyes et al. 2025b), while PTFE-NPLs effects were observed from 50 to 200 µg/mL in Caco-2 and HT29-MTX cells (Abass et al. 2025). For PET-NPLs, findings remain contradictory, with no effects reported in THP-1 cells up to 50 µg/mL for 3 h (Villacorta et al. 2022) but clear dose-dependent damage in A549 cells across starting from just 2 µg/mL (Alzaben et al. 2023). Taking together, this variability highlights that polymer chemistry, particle size, exposure dose, and cell type jointly shape the genotoxic potential of NPLs, and that absence of damage in one setting cannot be generalized across systems.

### Inflammation – IL-6 ELISA

Regarding the inflammatory response in HUVECs, IL-6 secretion was quantified in cell culture supernatants after 24 h exposure to NPLs (25 µg/mL). Exposure to PET-NPLs led to a significant increase in IL-6 secretion, reaching 1.93-fold relative to the control (*p* ≤ 0.001). This effect was also significantly higher compared with all other polymer types tested (*p* ≤ 0.01). In contrast, PS-NPLs, PLA-NPLs, and PTFE-NPLs did not induce significant changes in IL-6 levels compared with the control, indicating that the pro-inflammatory response was specific to real-life PET-NPLs under the tested conditions.

The absence of IL-6 induction after exposure to PS-NPLs, PLA-NPLs, and PTFE-NPLs is in line with several previous studies. For PS-NPLs, Weber et al. (2022) reported no increase in IL-6 when using monodisperse spherical particles of 50–310 nm in monocytes and dendritic cells, while Arribas Arranz et al. (2024) similarly observed no IL-6 response in whole blood at 100 µg/mL. In the case of PLA-NPLs, our results are consistent with da Silva et al. (2019), who found no pro-inflammatory activation in macrophages exposed to PLA-NPLs of comparable size across a wide dose range (0.5–100 µg/mL). For PTFE-NPLs, Abass et al. (2025) reported no induction of IL-8 secretion in an intestinal barrier model after 24 h exposure, further supporting the notion that PTFE-NPLs do not trigger acute pro-inflammatory signaling, at least through major cytokine and chemokine mediators. Together, these findings suggest that spherical, monodisperse polymers such as PS-NPLs, PLA-NPLs, and PTFE-NPLs generally exhibit limited pro-inflammatory activity under acute exposure conditions.

The enhanced IL-6 secretion triggered by real-life PET-NPLs is consistent with Weber et al. (2022) results, who reported that real-life, irregular, and polydisperse NPLs, including PS-NPLs and PVC-NPLs, induced IL-6 expression in monocytes and dendritic cells, whereas monodisperse spherical NPLs did not show such effects. These results suggest that particle morphology and colloidal behavior, rather than polymer identity alone, may play important roles in modulating inflammatory potential of NPLs.

**Fig. 4:**
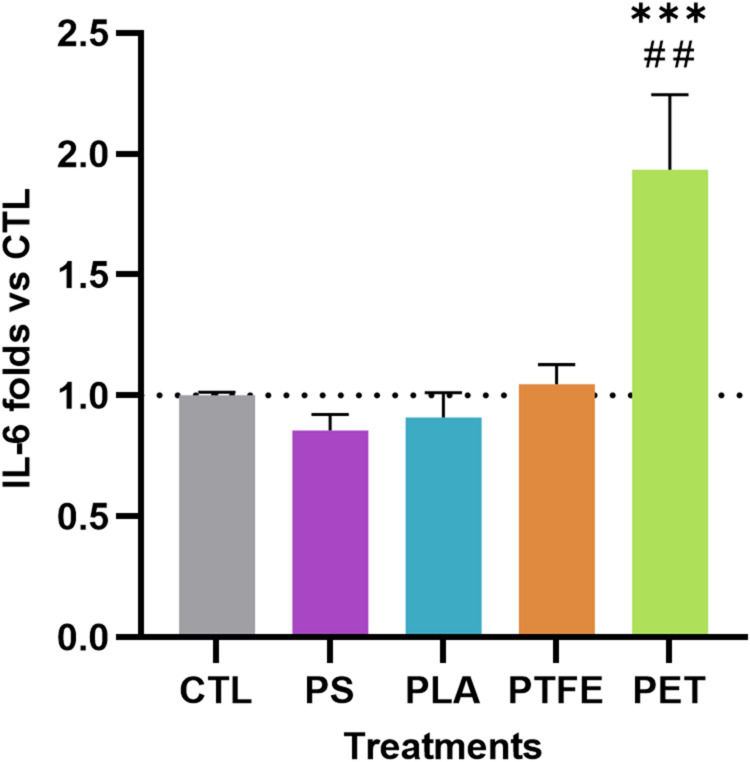
IL-6 secretion in HUVECs exposed to NPLs. IL-6 levels in culture supernatants were quantified by ELISA after 24 h exposure to NPLs (25 µg/mL). Bars represent fold change relative to the negative control (CTL, set to 1). Data are shown as mean ± SEM from ≥ 3 independent experiments. Statistical analysis was performed using one-way ANOVA followed by Dunnett’s test (comparisons vs. control) and Tukey’s test (pairwise comparisons among NPLs). ***p ≤ 0.001 vs. control; ^##^*p* ≤ 0.01 PET vs. different NPLs

The induction of IL-6 by PET-NPLs in HUVECs is biologically relevant because IL-6 is a central mediator of vascular inflammation and endothelial dysfunction (Kang et al. 2020). In endothelial cells, IL-6 signaling promotes inflammatory cell adhesion (Suzuki et al. 2010), disrupts endothelial barrier integrity increasing permeability and favoring vascular leakage (Kang et al. 2020), and induces a pro-thrombotic phenotype (Cimmino et al. 2022), collectively contributing to endothelial dysfunction and atherosclerosis development (Zegeye et al. 2023) (Fig. 4).

### Cholesterol detection

**Fig. 5:**
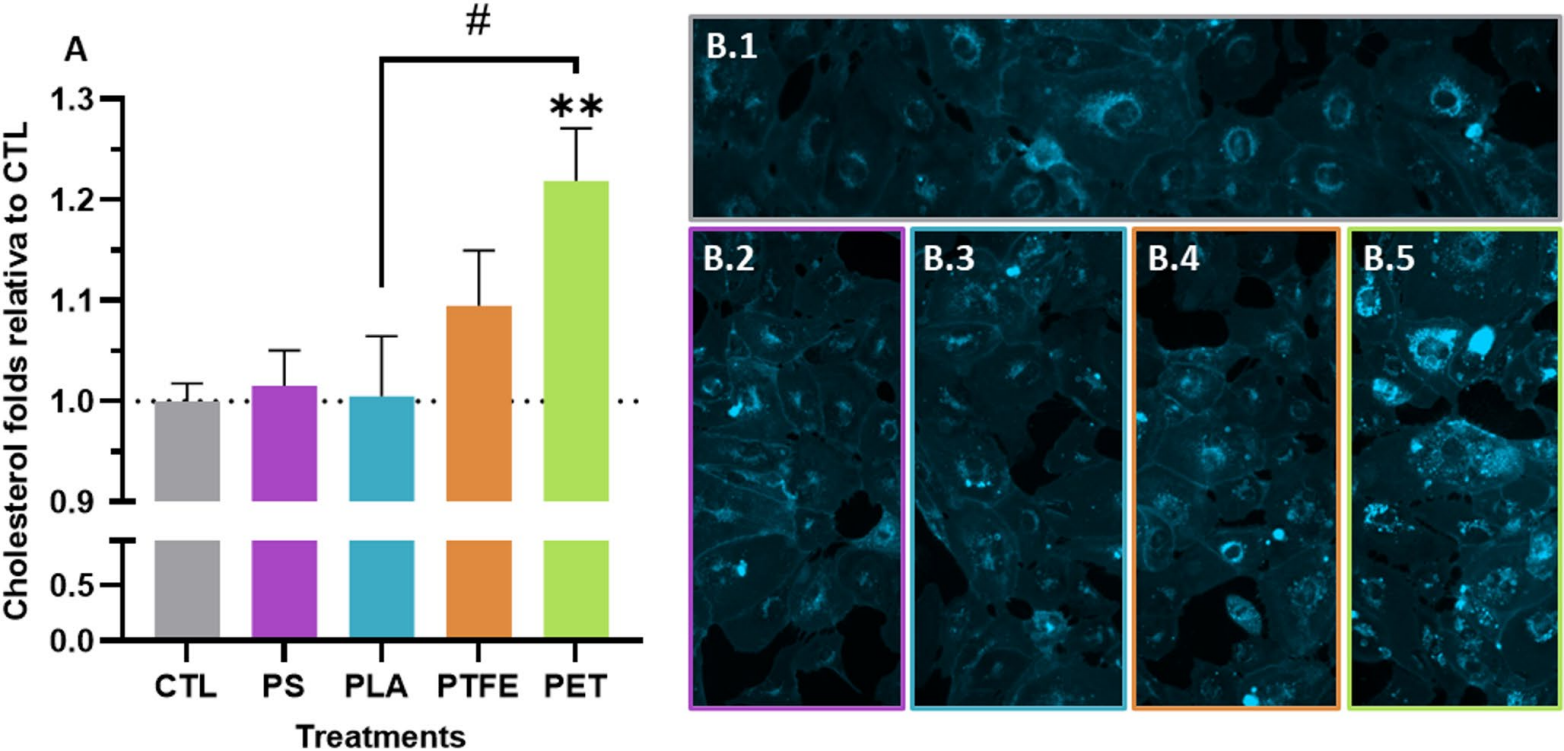
Cholesterol levels in HUVECs after NPLs exposure. **A** Cholesterol levels were quantified by Filipin III staining after 24 h exposure to NPLs (25 µg/mL). Bars represent fold change relative to the negative control (CTL, set to 1). Data are shown as mean ± SEM from ≥ 3 independent experiments. Statistical analysis was performed using one-way ANOVA followed by Dunnett’s test (comparisons vs. control) and Tukey’s test (pairwise comparisons among NPLs). **p ≤ 0.01 vs. control; ^#^*p* ≤ 0.05 PET-NPLs vs. PLA-NPLs. (B) Representative confocal images of Filipin III staining (cyan) in HUVECs exposed to **B.1** CTL, **B.2** PS-NPLs, **B.3** PLA-NPLs, **B.4** PTFE-NPLs, and **B.5** PET-NPLs. Images were acquired with a 20× objective

Filipin III staining was applied to visualize unesterified cholesterol within the cytoplasm of HUVECs. Fluorescence microscopy images (Figs. 5B.1–B.5) revealed clear differences across treatments, with PET-NPLs-treated cells (Fig. 5B.5) displaying visibly stronger fluorescence than control and the other polymers (Figs. 5B.1–B.4). Quantification of Filipin III intensity in approximately 600 individual cells confirmed these observations (Fig. 5A). After 24 h at 25 µg/mL, only PET-NPLs significantly increased intracellular cholesterol, reaching 1.22-fold compared with the negative control (*p* ≤ 0.01). PTFE-NPLs showed a modest but non-significant increase of 1.09-fold, whereas PS-NPLs and PLA-NPLs remained comparable to control. Pairwise comparisons indicated that PET-NPLs differed significantly only from PLA-NPLs (*p* ≤ 0.05).

PET-NPLs exposure also altered the spatial distribution of cholesterol. While control, PS-NPLs, PLA-NPLs, and PTFE-NPLs showed predominantly perinuclear fluorescence consistent with synthesis in the endoplasmic reticulum (Lyu et al. 2017), PET-NPLs resulted in a more dispersed cytoplasmic signal with punctate structures compatible with vesicular accumulation. Increased intracellular cholesterol has previously been reported in HUVECs exposed to PS-NPLs of smaller sizes up to 100 nm at higher concentrations of 100 µg/mL (Martín-Pérez et al. 2025). However, PS-NPLs did not induce significant changes in the present study, which may reflect the lower exposure concentration of 25 µg/mL or the larger particle size of 200 nm, since greater size is generally associated with reduced cellular reactivity (Płuciennik et al. 2024).

To our knowledge, the effects of NPLs beyond PS-NPLs on cholesterol metabolism have not yet been investigated. This study therefore provides the first evaluation of PLA-NPLs, PTFE-NPLs, and PET-NPLs on endothelial intracellular cholesterol. Only real-life PET-NPLs induced significant accumulation, likely due to physicochemical properties that favor the formation of unusually large vesicles up to 4 μm. Their persistence in the cytoplasm may impair vesicular trafficking pathways responsible for cholesterol export and redistribution, a process previously associated with elevated intracellular cholesterol levels (Litvinov et al. 2018; Martello et al. 2020). Interestingly, the cholesterol increase observed in PET-treated cells is also consistent with the pro-inflammatory response described above, as intermediates of cholesterol biosynthesis can promote IL-6 induction (Omoigui 2007).

Cholesterol accumulation is linked to endothelial dysfunction (Ziegler et al. 2024), whereas altered cholesterol trafficking affects membrane fluidity and cellular migration capacity (Lyu et al. 2017). In this context, we evaluated migration as a key component of endothelial function.

### Migration assay

**Fig. 6:**
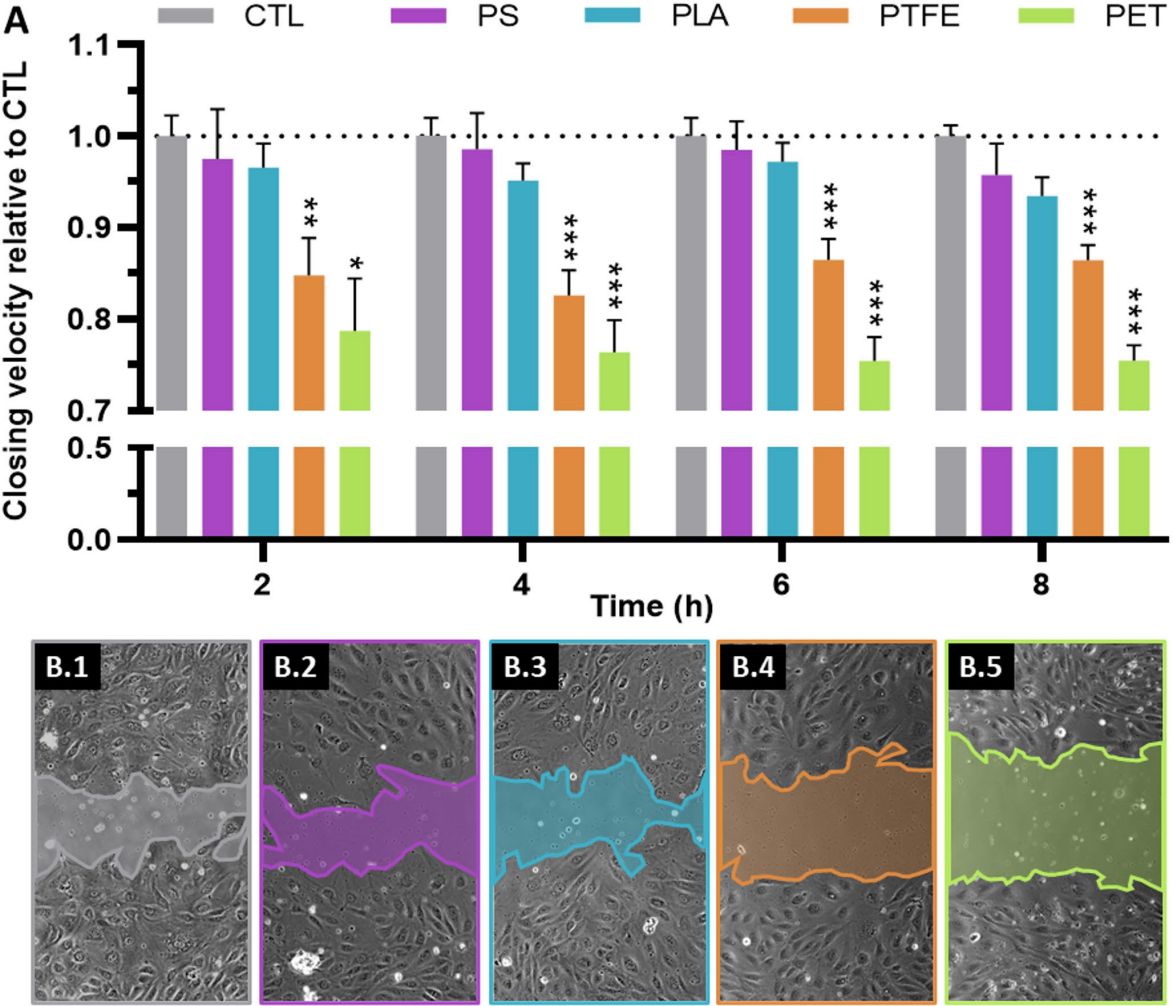
Effect of NPLs on HUVEC migration. **A** Closing velocity of HUVECs exposed for 24 h to NPLs (25 µg/mL) during wound healing assay, expressed as fold change relative to the negative control (CTL, set to 1). Data correspond to 2, 4, 6, and 8 h after scratch. Bars represent mean ± SEM from ≥ 4 independent experiments. Statistical analysis was performed by one-way ANOVA followed by Dunnett’s test (vs. control) and Tukey’s test (among NPLs). *p ≤ 0.05, **p ≤ 0.01, ***p ≤ 0.001. Complete results of pairwise comparisons for each time point are provided in Supplementary Table 1. **B** Representative phase-contrast images of wound areas at 8 h after scratch: **B.1** CTL, **B.2** PS-NPLs, **B.3** PLA-NPLs, **B.4** PTFE-NPLs, and **B.5** PET-NPLs. Colored lines delineate the wound edges used for quantification

The wound healing assay revealed polymer-specific effects on endothelial migration (Fig. 6). Closing velocity measurements (Fig. 6A) demonstrated that PS-NPLs and PLA-NPLs did not significantly alter HUVEC migration at any time point, whereas both PTFE-NPLs and PET-NPLs markedly impaired closure kinetics. PET-NPLs consistently induced the strongest inhibition, with migration velocity reduced by approximately 25% relative to the control at 8 h. The reduction was already significant from 2 h onwards and persisted throughout the assay. PTFE-NPLs produced a more moderate effect, leading to about a 15% decrease in closing velocity at 8 h, though this reduction was also significant and evident from the 2 h time-point. Pairwise analyses confirmed that PET-NPLs differed significantly from PS-NPLs and PLA-NPLs from 4 h onwards, and from PTFE-NPLs at 6–8 h. In addition, PTFE-NPLs differed significantly from PS-NPLs and PLA-NPLs starting at 4 h, except for the comparison with PLA-NPLs at 8 h, where no significant difference was detected (Supplementary Table S1). Representative phase-contrast images at 8 h illustrate the impaired wound closure in both PET-NPLs and PTFE-NPLs -treated cells compared with the efficient closure observed in control, PS-NPLs, and PLA-NPLs (Fig. 6B). These findings were reinforced by the complementary metric of wound closing percentage (Supplementary Fig. S5). The consistency across both readouts underscores the robustness of the observed impairment. Our results with PTFE-NPLs and PET-NPLs are in line with most of the existing research showing that MNPL exposure reduces cell migration under a variety of conditions. In vitro studies have consistently reported decreased migration in both HUVECs and human trophoblasts after exposure to PS-NPLs ranging from 30 to 500 nm (Lee et al. 2021; Hu et al. 2022; Lv et al. 2024; Wan et al. 2024). Comparable effects have also been demonstrated in vivo, where exposure of the freshwater leech *Hirudo verbana* to polypropylene MNPLs (100 nm–5 μm) delayed wound repair (Bon et al. 2025).

The inhibitory effects of PTFE-NPLs and PET-NPLs on migration may be linked to alterations in intracellular cholesterol levels, since elevated cholesterol in HUVECs has been associated with reduced migratory capacity (Lyu et al. 2017). This interpretation is consistent with our observations, with PET-NPLs inducing the highest intracellular cholesterol accumulation together with the most pronounced reduction in migration. Endothelial migration is essential for vascular repair, angiogenesis, and barrier integrity. Impairment of this process, as observed here for PTFE-NPLs and particularly PET-NPLs, can compromise angiogenesis and delay vascular repair, thereby aggravating pathological conditions such as atherosclerosis (Zhang et al. 2020). Taken together, our findings indicate that migration is a sensitive and functionally relevant endpoint for NPL toxicity in endothelial cells, with real-life polymers exerting the strongest inhibitory effects.

## Conclusions

This study provides a systematic comparison of NPLs of increasing environmental relevance, ranging from model spherical PS to irregular, polydisperse PET derived from post-consumer bottles, and their effects on primary human endothelial cells. By standardizing particle size and exposure conditions, we were able to assess how increasing environmental significance, defined by particle morphology, size heterogeneity, and colloidal behavior, influences endothelial responses. All NPLs were efficiently internalized by HUVECs, although their intracellular distribution varied, suggesting the involvement of distinct uptake pathways depending on their physicochemical characteristics. None of the NPLs induced cytotoxicity or genotoxicity after 24 h of exposure at 25 µg/mL. However, functional assays revealed clear differences related to NPL properties. Spherical and monodisperse PS- and PLA-NPLs produced negligible alterations, whereas irregular, heterogeneous, and polydisperse PTFE- and PET-NPLs triggered functionally relevant signs of endothelial stress. Specifically, PET-NPLs promoted IL-6 secretion and intracellular cholesterol accumulation, while both PTFE- and PET-NPLs significantly reduced cell migration, compromising the wound-healing capacity of endothelial monolayers. The observed gradient of biological responses supports the notion that particle shape, heterogeneity, and colloidal behavior are key determinants of NPL bioactivity. Altogether, our findings establish a potential link between real-life NPLs and early vascular impairment through inflammation, cholesterol dysregulation, and migration inhibition. They also emphasize the limitations of relying exclusively on idealized, monodisperse NPLs for hazard assessment. Incorporating environmentally realistic NPLs into toxicological testing is essential to predict genuine human health risks and to design regulatory frameworks that capture the true complexity of plastic pollution. To extend this work, future studies should include NPLs with a broader diversity of shapes, such as fibers or fragments, and employ more advanced experimental systems, particularly endothelial barriers and microfluidic tissue-engineered vascular models to better reproduce physiological conditions. Beyond these controlled in vitro settings, addressing more complex atherosclerosis-related outcomes that are outside the scope of short-term static HUVEC monocultures (e.g., plaque formation and stenosis-related processes), will ultimately require in vivo studies and human biomonitoring approaches to capture systemic, long-term and flow-dependent mechanisms. Such approaches may refine our understanding of NPL-induced vascular dysfunction and its potential cardiovascular implications.

## Supplementary Information

Below is the link to the electronic supplementary material.

Supplementary Material 1
