# Supplementary material for: Real-life nanoplastics induce endothelial dysfunction in primary human endothelial cells
Source: Arch Toxicol. 2026 May 5;100(8):3377–93. doi: 10.1007/s00204-026-04417-9 (PMC13379464; doi:10.1007/s00204-026-04417-9)
Supplement: Supplementary file 1 — Supplementary Material 1 [file 204_2026_4417_MOESM1_ESM.docx]

**Real-life nanoplastics induce endothelial dysfunction in primary human endothelial cells**

**Joan Martín-Pérez^1^, Michelle Morataya-Reyes^1^, Aliro Villacorta^1,2^, Claudia Anguita-Solé^1^, Juan Francisco Ferrer^3^**, **Irene Barguilla^1^, Mohamed Alaraby^1,4^, Ricard Marcos^1^, Alba Hernández^1,*^, Alba García-Rodríguez^1,*^**

^1^*Group of Mutagenesis, Department of Genetics and Microbiology, Faculty of Biosciences, Universitat Autònoma de Barcelona, 08193 Cerdanyola del Vallès, Spain.*

^2^*Facultad de Recursos Naturales Renovables, Universidad Arturo Prat,101000 Iquique, Chile.*

*^3^AIMPLAS, Plastics Technology Center, Valencia Parc Tecnològic, 46980 Paterna, Spain.*

*^4^Zoology Department, Faculty of Science, Sohag University (82524), Sohag, Egypt.*

**SUPPLEMENTARY MATERIAL**


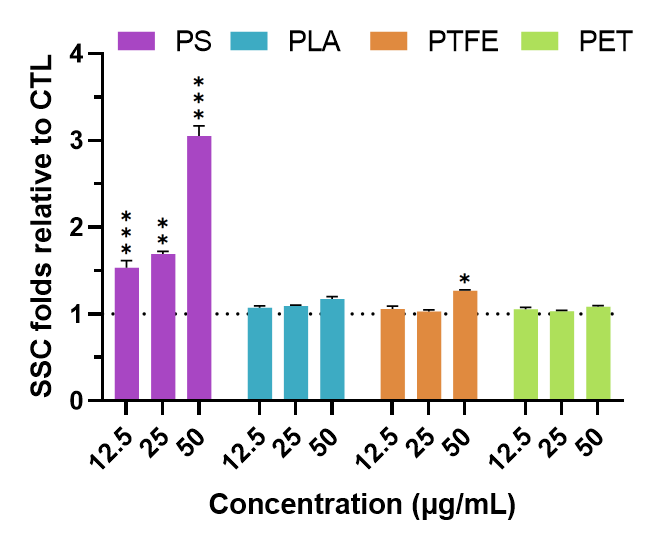


**Figure S1.** Effect of NPLs on cell complexity in HUVECs. Side scatter (SSC) was measured by flow cytometry as an indicator of cell granularity/complexity after 24 h exposure to increasing concentrations of NPLs (12.5, 25, and 50 µg/mL). Bars represent mean ± SEM from ≥3 independent experiments. Statistical analysis was performed using the Kruskal–Wallis test followed by Dunn’s multiple comparisons test *vs* the negative control (CTL) for each concentration. **p* ≤ 0.05, ***p* ≤ 0.01, ****p* ≤ 0.001.


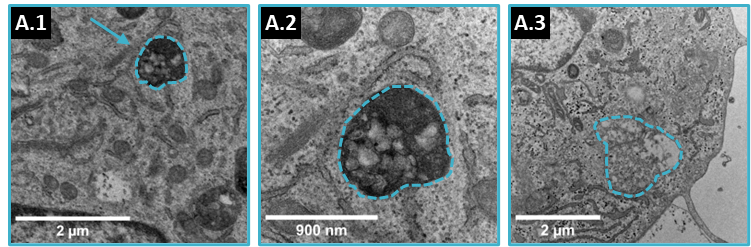


**Figure S2.** Internalization of PLA-NPLs in HUVECs. (A.1) TEM image at lower magnification showing the cytoplasm of a HUVEC exposed to 25 µg/mL PLA-NPLs for 24 h. The blue arrow indicates an intracellular vesicle containing nanoparticles. (A.2) Higher magnification of the vesicle highlighted in (A.1), outlined with a blue dashed line, with an approximate diameter of 700-800 nm. Inside, multiple particles of 100–200 nm are observed, consistent with internalized PLA-NPLs.


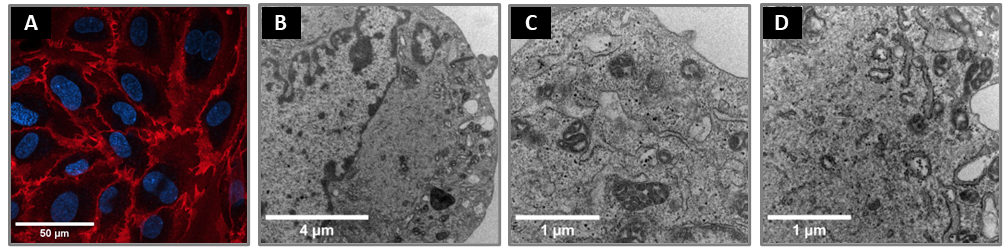


**Figure S3.** Negative controls of HUVECs. (A) Representative confocal fluorescence image of untreated HUVECs showing nuclei (blue) and cell membrane (red). No green signal corresponding to NPLs is detected. (B–D) Transmission electron microscopy (TEM) images of untreated HUVECs displaying normal ultrastructural morphology without evidence of nanoparticle internalization.

**Figure S4.** Basal DNA damage (single-strand breaks) in HUVECs exposed to NPLs. Basal DNA damage was assessed by the comet assay without FPG after 24 h of exposure to NPLs (25 µg/mL). Data are expressed as fold change relative to the negative control (CTL, set to 1); bars represent mean ± SEM of ≥2 independent experiments. Methyl methanesulfonate (MMS, 200 µM, 40 min) was used as a positive control for single-strand breaks. Statistical analysis was performed using the Kruskal–Wallis test with the . Significant differences were observed only between positive control and negative control; no significant differences were detected for NPLs *vs* control or among NPLs. ****p* ≤ 0.001.


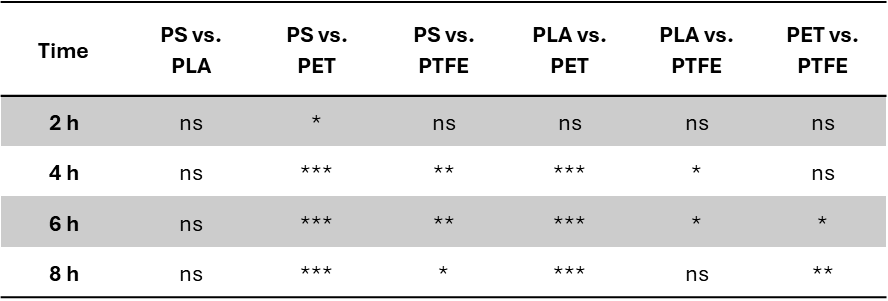


**Table S1.** Pairwise multiple comparisons of closing velocity among NPL treatments at different exposure times (2–8 h). Statistical significance was assessed by the one-way ANOVA followed by Tukey’s post hoc test. ns = not significant; **p* ≤ 0.05, ***p* ≤ 0.01, ****p* ≤ 0.001.


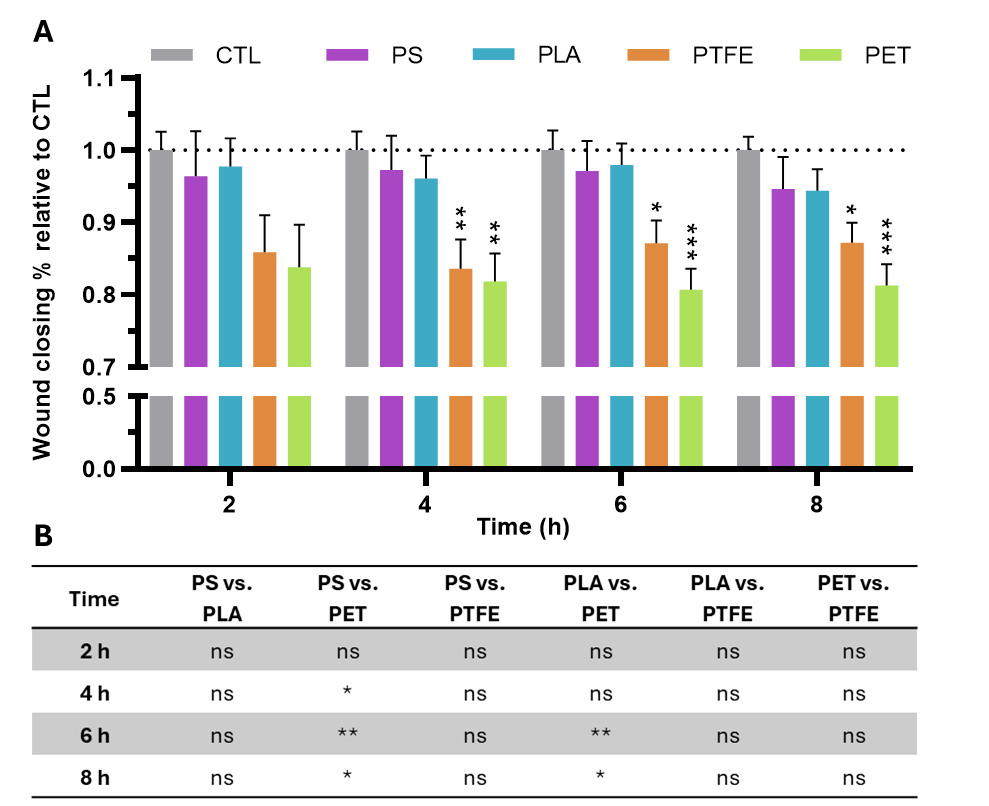


**Figure S5.** Effect of NPLs on HUVEC wound healing. (A) Wound closing percentage of HUVECs exposed for 24 h to NPLs (25 µg/mL), expressed as fold change relative to the negative control (CTL, set to 1). Data corresponds to 2, 4, 6, and 8 h after scratch. Bars represent mean ± SEM from ≥4 independent experiments. (B) Table summarizing multiple pairwise comparisons among treatments for each time point. Statistical analysis was performed by one-way ANOVA followed by Tukey’s test or, when data did not meet parametric assumptions, by Kruskal–Wallis followed by Dunn’s test. ns = not significant; **p* ≤ 0.05, ***p* ≤ 0.01,
